# Drug use and COVID-19 testing, vaccination, and infection among underserved, minority communities in Miami, Florida

**DOI:** 10.1371/journal.pone.0297327

**Published:** 2024-04-30

**Authors:** Javier A. Tamargo, Haley R. Martin, Janet Diaz-Martinez, Ivan Delgado-Enciso, Angelique Johnson, Jose A. Bastida Rodriguez, Mary Jo Trepka, David R. Brown, Nana A. Garba, Eneida O. Roldan, Yolangel Hernandez Suarez, Aileen M. Marty, Zoran Bursac, Adriana Campa, Marianna K. Baum

**Affiliations:** 1 Robert Stempel College of Public Health & Social Work, Florida International University, Miami, Florida, United States of America; 2 Faculty of Medicine, University of Colima, Las Víboras, Colima, Mexico; 3 Cancerology State Institute, Colima State Health Services, La Esperanza, Colima, Mexico; 4 Herbert Wertheim College of Medicine, Florida International University, Miami, Florida, United States of America; Universitas Syiah Kuala, INDONESIA

## Abstract

The Coronavirus Disease 2019 (COVID-19) pandemic has disproportionately impacted people who use drugs (PWUD). This study explored relationships between drug use, COVID-19 testing, vaccination, and infection. This cross-sectional study was conducted in Miami, Florida between March 2021 and October 2022 as part of the National Institutes of Health (NIH) Rapid Acceleration of Diagnostics-Underserved Populations (RADx-UP) initiative and the Miami Adult Studies on HIV (MASH) cohort. Users of cannabis, cocaine/crack, heroin/fentanyl, methamphetamines, hallucinogens, and/or prescription drug misuse in the previous 12 months were considered PWUD. Sociodemographic data, COVID-19 testing history, and vaccination-related beliefs were self-reported. Vaccinations were confirmed with medical records and positivity was determined with severe acute respiratory syndrome coronavirus 2 (SARS-CoV-2) testing. Statistical analyses included chi-square tests and logistic regression. Of 1,780 participants, median age was 57 years, 50.7% were male, 50.2% Non-Hispanic Black, and 66.0% reported an annual income less than $15,000. Nearly 28.0% used drugs. PWUD were less likely than non-users to self-report ever testing positive for SARS-CoV-2 (14.7% vs. 21.0%, *p* = 0.006). However, 2.6% of participants tested positive for SARS-CoV-2, with no significant differences between PWUD and non-users (3.7% vs. 2.2%, *p* = 0.076). PWUD were more likely than non-users to experience difficulties accessing testing (10.2% vs. 7.1%, *p* = 0.033), vaccine hesitancy (58.9% vs. 43.4%, *p* = 0.002) and had lower odds of receiving any dose of a COVID-19 vaccine compared to non-users (aOR, 0.63; 95% CI, 0.49–0.81; *p*<0.001). PWUD presented with greater difficulties accessing COVID-19 testing, greater vaccine hesitancy, and lower odds of vaccination. Testing and immunization plans that are tailored to the needs of PWUD and consider access, trust-building campaigns, and education may be needed.

## Introduction

The Coronavirus Disease 2019 (COVID-19) pandemic has disproportionately impacted people who use drugs (PWUD), who are among the most socially vulnerable populations [1–3]. Social vulnerability refers to the negative effects of external stressors on the health of communities; stressors such as lack of income, limited transportation and healthcare access, as well as housing insecurity may weaken a community’s ability to prepare for and respond to hazardous events [4]. Indeed, PWUD are more likely to live in poverty [5], experience housing instability, and have difficulty accessing healthcare [6]. Over 20% of the United States (U.S.) population use illicit substances [7], and the COVID-19 pandemic has led to exacerbation of substance use [8, 9].

Despite the effectiveness of COVID-19 vaccination in preventing severe illness, hospitalization, and death due to COVID-19 [10], vaccination rates remain low among U.S. minorities [11]. Barriers to vaccination may include poor access to vaccination sites, fear of mistreatment by medical professionals, and mistrust of the medical community [12, 13]. Sources have reported hesitancy rates of approximately 19.8% in the U.S. [14], while literature on global COVID-19 vaccine acceptance and hesitancy have reported hesitancy rates as high as 33.0% in the U.S. [15]. PWUD may also encounter barriers that limit vaccination access such as lack of transportation [16], limited healthcare access [6], concerns about stigma and discrimination [17], and complexity of booking systems [18, 19]. In a survey of PWUD, nearly half expressed unwillingness to take a COVID-19 vaccine, citing concerns of adverse effects and skepticism of risk of infection [20]. Studies investigating people who inject drugs have indeed reported lower COVID-19 vaccination rates in Australia and Baltimore, Maryland in the U.S. [18, 21]. Reports of lower flu vaccine [22, 23] and hepatitis A and B vaccine uptake [24, 25], as well as low willingness for a potential HIV vaccine uptake among PWUD have also been published [26]. Nevertheless, data regarding COVID-19 vaccine uptake among PWUD in the Southern U.S. is lacking.

Studies have reported PWUD are at an increased risk of severe COVID-19 illness, hospitalization, and mortality [27–29], potentially due to the same structural and social factors that contribute to the community’s social vulnerability. PWUD may also be at risk of poor COVID-19 outcomes due to underlying cardiometabolic comorbidities and pulmonary damage caused by use of illicit substances [28–31]. Many drugs of abuse also have immunosuppressive properties that may promote infection and disease progression [32, 33]. Opioids have been shown to exert suppressive effects on both the innate and adaptive immune systems [34], while methamphetamines have been reported to cause immune dysregulation in the lungs [35]. Smoking of cannabis can damage lung tissue leading to inflammation and diminished capacity to respond to respiratory infections [30], while cocaine, methamphetamines, and opioids have all been reported to negatively affect the cardiovascular system [28]. Altogether, many drugs of abuse, including cannabis which has been reported to be more socially acceptable in the U.S. [36], may have adverse physiological effects that may increase the risk for poor COVID-19 outcomes. Taken together with the social vulnerability that accompanies drug use [1], strategies are needed to monitor and prevent the spread of COVID-19 among PWUD.

COVID-19 testing, another critical component in controlling the spread of COVID-19, has also not been well investigated among PWUD. In this study, we explored relationships between drug use, COVID-19 testing, vaccination, and infection.

## Materials and methods

### Study design

This cross-sectional study was conducted as part of the National Institutes of Health (NIH) Rapid Acceleration of Diagnostics-Underserved Populations (RADx-UP) initiative; a consortium of more than 135 projects studying COVID-19 testing patterns in underserved communities across the U.S. The overall aim of RADx-UP is to investigate COVID-19 testing patterns in historically marginalized and underserved communities across the U.S. with the aim of speeding innovation in the development and implementation of testing. More information can be found at https://radx-up.org/about/. The data presented herein were collected from an individual RADx-UP project site located in Miami, Florida as part of RADx-UP Phases I and II between March 30, 2021 and October 13, 2022. Phase I recruited participants and collected data from March 2021 to February 2022 and Phase II then recruited participants and collected data from May 2022 to October 2022. Both our Phase I and II projects collected the same variables, and each Phase recruited new participants. Importantly, Miami-Dade County experiences a high level of social vulnerability [37]; the COVID-19 Community Vulnerability Index scored Miami Dade as 0.90 which indicates “very high” COVID-19 vulnerability [38]. Recruitment for this RADx-UP project site included community members from socioeconomically disadvantaged Black and Hispanic neighborhoods and participants of the Miami Adult Studies on HIV (MASH) cohort; a prospective cohort study funded by the National Institutes on Drug Abuse (NIDA) that follows more than 1,000 underserved minority Black and Hispanic adults living with and without HIV and high rates of substance use [39]. The protocol for this study was approved by the Institutional Review Board (IRB) at Florida International University (FIU). The authors had access to information that could identify individual participants during and after data collection; IRB-approved privacy and confidentiality protocols were adhered to. The data were accessed for this study between September 2021 and June 2023. All participants provided verbal informed consent to participate in the study over the phone which was witnessed and documented by two trained research assistants. Written consent was not obtained due to COVID-19 precautionary measures, which was approved by the FIU IRB.

### Data collection

Due to COVID-19 precautionary measures, participants completed questionnaires by phone with a trained interviewer. Exceptions for in-person questionnaires were made for participants without access to a phone or with difficulty communicating over the phone. A screening consisted of sociodemographic and eligibility questions. Inclusion criteria consisted of being 18 years of age and older. Participants were excluded if they were pregnant, unwilling to complete the survey, and/or unwilling to undergo COVID-19 testing. If eligible, participants completed the survey which included RADx-UP common data elements (CDEs) and additional questions implemented by our project site which included measures of substance use behaviors, comorbidities, and health disparities. After participants completed the survey over the phone, they were provided an appointment for COVID-19 testing at our clinic, located in central Miami, Florida. Participants were tested for severe acute respiratory syndrome coronavirus 2 (SARS-CoV-2) with real-time reverse transcription-polymerase chain reaction (rt-PCR) in our Clinical Laboratory Improvement Amendments (CLIA)-certified COVID-19 lab. Medical personnel performed sampling, with participant’s choice of nasopharyngeal swab or saliva. Participants were compensated $15 for completing the screening and $40 for completing the survey with COVID-19 testing.

The RADx-UP CDEs include items from the NIH CDE Repository, Disaster Research Response (DR2) guidelines, and the PhenX Toolkit. The RADx-UP consortium selected and further refined community-informed CDEs via an iterative process to support standardized data collection [40]. The RADx-UP CDEs and sources are available at https://radx-up.org/. Sociodemographic data, alcohol and tobacco use, COVID-19 testing history and related beliefs, and COVID-19 vaccination-related beliefs were self-reported. Employment status was assessed with a PhenX protocol item, “We would like to know about what you do—are you working now, looking for work, retired, keeping house, a student, or something else?”. A response of, “Disabled, permanently or temporarily” was used to denote disabled employment status which differed from an unemployed status which was defined as a response of, “Only temporarily laid off, sick leave or maternity leave”, or “Looking for work, unemployed”. COVID-19 status at the time of the study was determined with rt-PCR testing for SARS-CoV-2 as described above. COVID-19 vaccinations and HIV status were confirmed with medical record documentation. We confirmed 81.8% of all self-reported COVID-19 vaccines by compensating participants an additional $5 for a copy of their vaccination record card. HIV viral load and CD4 cell counts were abstracted from medical records for participants living with HIV, following consent.

### PWUD

We use the term “PWUD” to refer to people who use illicit substances including cannabis and misuse of prescription drugs. Participants were asked about their use of marijuana in the past 12 months (yes, no). Our rationale for including cannabis was that it remains illicit at the federal level, can be abused, and there exists ample evidence of the possibility of cannabis dependence [41]. Cannabis remains the most widely used illicit drug [7], and poor health outcomes have been observed even among people who use marijuana for at least some therapeutic purposes [42]. Misuse of prescription drugs was assessed by asking, “In the past 12 months, how often have you used prescription drugs just for the feeling, more than prescribed, or that were not prescribed for you?” (never, rarely, about once per month, about once or twice per week, daily or almost daily); misuse of prescription drugs was considered as, “about once per month” or more frequently. Lastly, participants were asked, “In the past 12 months, have you used any of the following drugs: cocaine or crack, heroin, fentanyl, crystal meth (methamphetamine), hallucinogens (like LSD, psilocybin, PCP, ketamine), ecstasy?” (yes, no).

### Statistical analysis

Descriptive statistics are presented as counts (percent, %) for categorical variables and mean ± standard deviation (SD) or median (Q1 –Q3) for continuous variables depending on the normality of the distribution of the variable which was assessed with the Shapiro-Wilk test and examination of histograms. Between-group differences were tested with chi-square test and t-tests (Wilcoxon rank-sum test for variables with skewed distributions) for categorical and continuous outcomes, respectively. The main exposure of interest was drug use. The primary outcomes were COVID-19 testing, vaccination, and infection. Binary logistic regressions were performed and reported as prevalence odds ratios (OR) with 95% confidence intervals (CI). Multivariable regression models adjusted for age, sex, race/ethnicity, education level, and history of lung disease. COVID-19 vaccination status was also included as a covariate for the regression models with COVID-19 positivity as the outcome. The variance inflation factor method was used to ensure absence of collinearity between independent variables. Missing data were treated as missing at random and were excluded from respective analyses, as previously suggested [43]. Results were considered statistically significant at two-tailed p<0.05. All statistical analyses were conducted using SAS OnDemand for Academics (SAS, Inc. Cary, NC).

## Results

### Cohort characteristics

**Fig 1:**
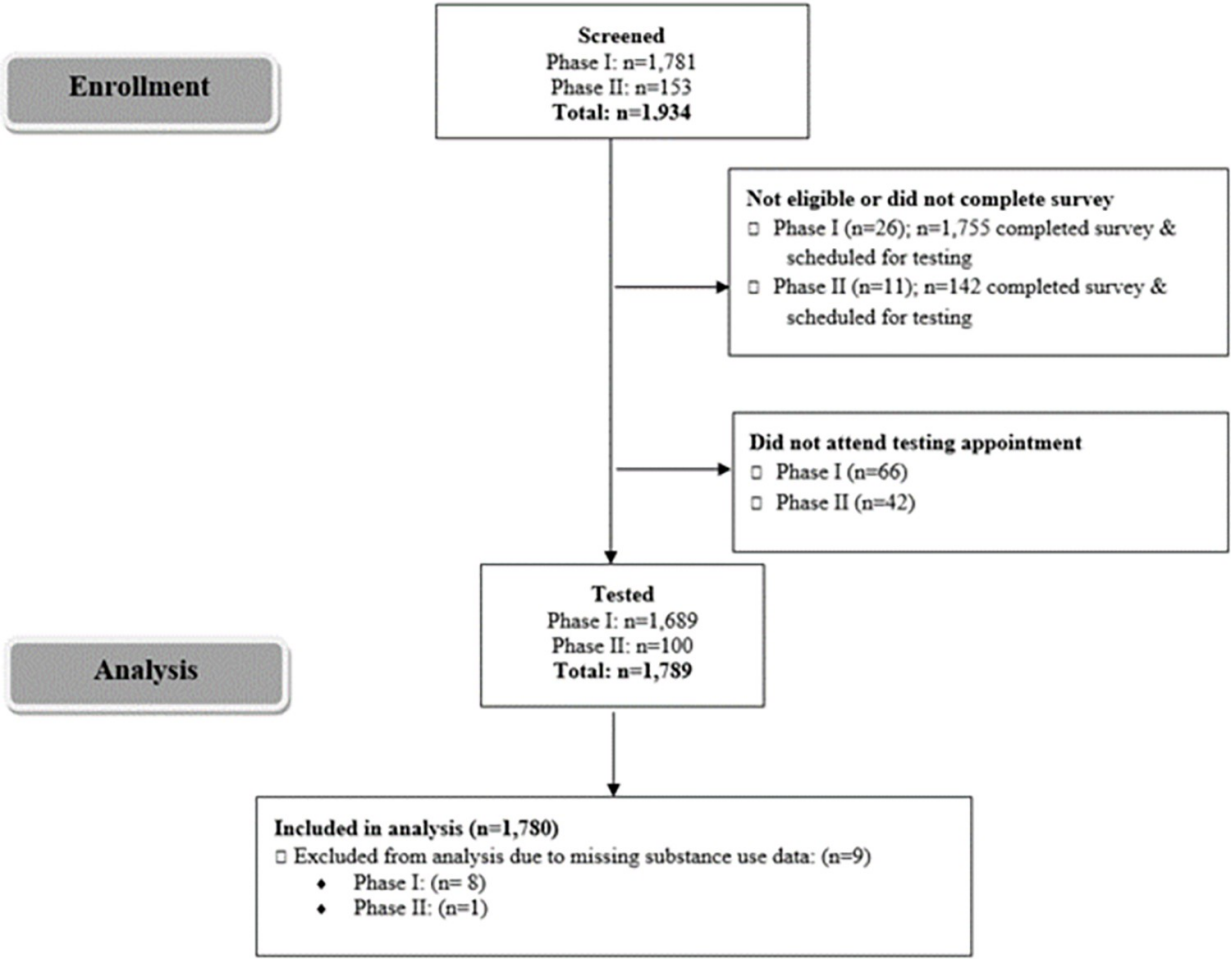
Flow diagram of enrollment and testing of RADx-UP study participants. A visualization of the flow of screening, exclusion, enrollment, testing, and final sample size of the analysis. Abbreviations: RADx-UP, Rapid Acceleration of Diagnostics–Underserved Populations.

**Fig 2:**
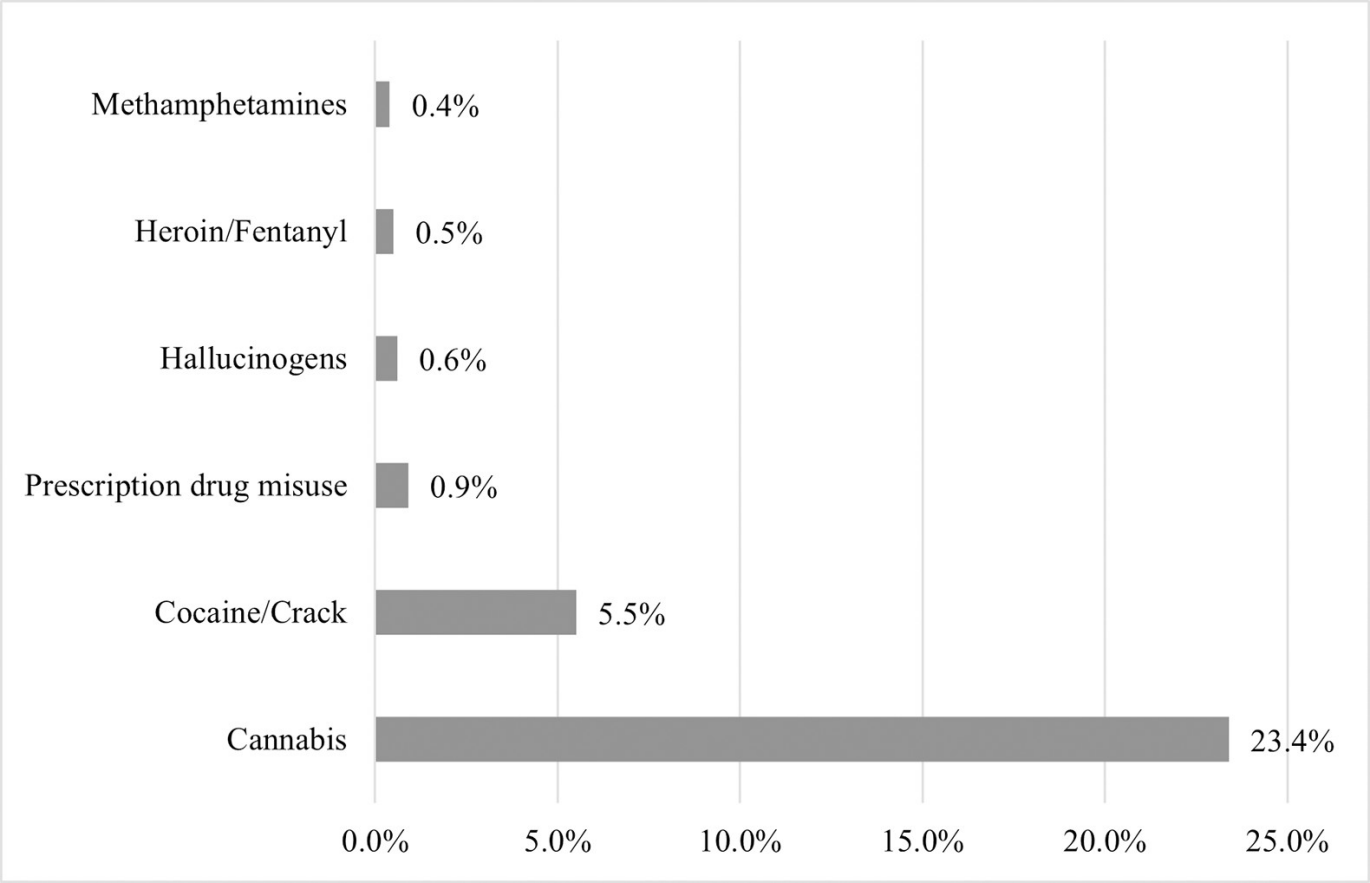
Percent of RADx-UP participants who reported substance use stratified by type of substance, Miami, Florida, March 2021 –October 2022. A visualization of the percentage of participants who reported substance use stratified by type of substance. Cannabis use was defined as a response of “yes” to “Have you used marijuana in the past 12 months?”. Cocaine/crack use, hallucinogen use, heroin/fentanyl use, and methamphetamine use was defined as a response of “About once per month”, “About once or twice per week”, or “Daily or almost daily” to “In the past 12 months, have you used any of the following drugs: cocaine or crack, heroin, fentanyl, crystal meth (methamphetamine), hallucinogens (like LSD, psilocybin, PCP, ketamine), ecstasy?” for each of the individual drugs. Prescription drug misuse was defined as a response of “About once per month”, “About once or twice per week”, or “Daily or almost daily” to “In the past 12 months, how often have you used prescription drugs just for the feeling, more than prescribed, or that were not prescribed for you?”. Abbreviations: RADx-UP, Rapid Acceleration of Diagnostics–Underserved Populations.

A total of 1,781 and 153 RADx-UP participants were screened for possible inclusion into Phases I and II, respectively. Participants were excluded from analysis due to ineligibility or unwillingness to complete the survey, not attending their testing appointment, and/or missing substance use data (Fig 1). A total of 1,681 participants from Phase I and 99 participants from Phase II were included (n = 1,780); 533 (29.9%) were MASH cohort participants. Nearly 28.0% reported using drugs, including cannabis (23.4%), cocaine/crack (5.5%), misuse of prescription drugs (0.9%), hallucinogens (0.6%), heroin and/or fentanyl (0.5%), and methamphetamines (0.4%) (Table 1, Fig 2). Due to the small proportions (<1%) of those who reported use of drugs other than cannabis or cocaine/crack, we were unable to appropriately conduct analyses stratified by each drug type separately. Thus, we report comparisons between those who reported use of any illicit drug vs. those who reported no drug use. Additionally, 35.8% smoked cigarettes, 4.5% consumed alcohol four or more days per week, and 10.3% engaged in binge drinking. Compared to those who did not use drugs, PWUD were more likely to smoke (27.2% vs.58.6%, *p*<0.001), engage in frequent alcohol use (2.0% vs.10.0%, *p*<0.001), and binge drink (7.3% vs. 15.5%, *p*<0.001) (Table 1).

**Table 1:** Characteristics of RADx-UP study participants by drug use status.

Variable ^a^		Total	Non-Users	People Who Use Drugs ^b^	*p*
**Total**		n = 1,780	n = 1,290 (72.5)	n = 490 (27.5)	
**Age**		57.0 (49.0–63.0)	58.0 (50.0–64.0)	56.0 (43.0–61.0)	<0.001 *
**Sex assigned at birth**	Male	903 (50.7)	597 (46.3)	306 (62.5)	<0.001 *
**Race/Ethnicity**	Black non-Hispanic	890 (50.2)	569 (44.3)	321 (65.9)	<0.001 *
	Black Hispanic	98 (5.5)	78 (6.1)	20 (4.1)	
	White non-Hispanic	102 (5.8)	61 (4.8)	41 (8.4)	
	White Hispanic	617 (34.8)	532 (41.4)	85 (17.4)	
	Other	65 (3.7)	45 (3.5)	20 (4.1)	
**Education**	Less than high school	605 (34.1)	438 (34.0)	167 (34.1)	<0.001 *
	High school or GED	521 (29.3)	354 (27.5)	167 (34.1)	
	Some college	425 (23.9)	295 (22.9)	130 (26.5)	
	College degree	226 (12.7)	200 (15.5)	26 (5.3)	
**Household**	Number in household	2 (1–3)	2 (1–3)	2 (1–3)	0.145
	Lives alone	712 (40.0)	502 (38.9)	210 (42.9)	0.010 *
	Lives with spouse	306 (17.2)	230 (17.8)	76 (15.5)	
	Lives with children/multi-generational home	462 (26.0)	356 (27.6)	106 (21.6)	
	Lives with none of these/other type of household	300 (16.9)	202 (15.7)	98 (20.0)	
**Unstable housing**	In the past two months, have you been staying in the same place? (No)	144 (8.1)	99 (7.7)	45 (9.2)	0.294
**Housing insecure**	Are you worried or concerned that in the next two months you may NOT have a place to stay? (Yes)	248 (14.0)	143 (11.2)	105 (21.9)	<0.001 *
**Employment**	Working now	384 (21.7)	290 (22.6)	94 (19.2)	<0.001 *
	Unemployed ^c^	400 (22.6)	271 (21.1)	129 (26.4)	
	Retired	233 (13.1)	194 (15.1)	39 (8.0)	
	Disabled ^d^	656 (37.0)	454 (35.3)	202 (41.3)	
	Other ^e^	101 (5.7)	76 (5.9)	25 (5.1)	
	Have you, or has anyone in your household, experienced a loss of employment income since the start of the COVID-19 pandemic (March 2020)? (Yes)	564 (31.7)	373 (28.9)	191 (39.0)	<0.001 *
**Health insurance**	None/Don’t know	333 (18.8)	209 (16.3)	124 (25.3)	<0.001 *
	Private	260 (14.7)	215 (16.8)	45 (9.2)	
	Public	1,176 (66.5)	855 (66.9)	321 (65.5)	
**Annual household income in 2019**	Less than $15,000	1,174 (66.0)	827 (64.2)	347 (70.8)	0.0871
	$15,000 –$34,999	398 (22.4)	299 (23.2)	99 (20.2)	
	$35,000 –$74,999	108 (6.1)	85 (6.6)	23 (4.7)	
	$75,000 and more	23 (1.3)	19 (1.5)	4 (0.8)	
	*Prefer not to answer*	76 (4.3)	59 (4.6)	17 (3.5)	
**Frequent alcohol use**	Drinks alcohol 4 times a week or more (n = 1,547) ^f^	69 (4.5)	22 (2.0)	47 (10.0)	<0.001 *
**Binge drinking**	Drinks 5 or more standard alcoholic drinks when drinking (n = 982) ^g^	101 (10.3)	46 (7.3)	55 (15.5)	<0.001 *
**Tobacco use**	Never smoker	771 (43.4)	660 (51.2)	111 (22.7)	<0.001 *
	Former smoker	369 (20.8)	277 (21.5)	92 (18.8)	
	Some days smoker	166 (9.3)	93 (7.2)	73 (14.9)	
	Every day smoker	472 (26.6)	258 (20.0)	214 (43.7)	
**Living with HIV**		403 (22.6)	267 (20.7)	136 (27.8)	0.002 *
	On antiretroviral therapy	380 (96.5)	255 (97.0)	125 (95.4)	0.564
	Virally suppressed (n = 238) ^h^^,^^i^	200 (84.0)	131 (84.5)	69 (83.1)	0.781
	CD4 lymphocyte count, cells/μL (n = 253) ^j^	564.0 (383.0–849.0)	543.0 (383.0–858.0)	569.0 (383.0–838.0)	0.777

Data are presented as count (percent, %) for categorical variables, mean ± standard deviation (SD) for normally distributed continuous variables, and median (interquartile range) for continuous variables with a skewed distribution.

Reported use of cannabis, cocaine or crack, heroin, fentanyl, methamphetamine, hallucinogens, ecstasy, or misuse of prescription drugs in the past 12 months.

Defined as a response of, “Only temporarily laid off, sick leave or maternity leave”, or “Looking for work, unemployed” to a PhenX protocol item (PhenX PX011301) designed to assess employment status, “We would like to know about what you do—are you working now, looking for work, retired, keeping house, a student, or something else?”.

Defined as a response of, “Disabled, permanently or temporarily” to a PhenX protocol item (PhenX PX011301) designed to assess employment status, “We would like to know about what you do—are you working now, looking for work, retired, keeping house, a student, or something else?”.

Defined as a response of, “Keeping house”, “Student” or “Other” to a PhenX protocol item (PhenX PX011301) designed to assess employment status, “We would like to know about what you do—are you working now, looking for work, retired, keeping house, a student, or something else?”.

Item asked to participants who responded “yes” to “In your entire life, have you had at least 1 drink of any kind of alcohol, not counting small tastes or sips?”.

Item asked to participants who reporting drinking alcohol more than “never”.

Virally suppressed was defined as <200 copies of HIV per mL of blood.

HIV viral load data abstracted from medical records; values within four months of the participants’ visit to our clinic were considered.

CD4 lymphocyte count data abstracted from medical records; values within four months of the participants’ visit to our clinic were considered.

* p-value <0.05

Abbreviations: CD4, cluster of differentiation 4; RADx-UP, Rapid Acceleration of Diagnostics–Underserved Populations

The median age of participants was 57.0 (49.0–63.0), 50.7% were male, 50.2% identified as Non-Hispanic Black, 34.8% as Hispanic White, and 5.8% as Non-Hispanic White; 63.3% obtained a high school education or less, 37.0% were disabled, 22.6% were living with HIV, and 21.7% were employed (Table 1). Compared to those who did not use drugs, PWUD were more likely to be slightly younger, male, Non-Hispanic Black, and living with HIV, and less likely to have a college degree. PWUD were also more likely to live alone (42.9% vs. 38.9%, *p* = 0.010), report housing insecurity (21.9% vs. 11.2%, *p*<0.001), employment disability (41.3% vs. 35.3%, *p*<0.001), and loss of income since the start of the COVID-19 pandemic (39.0% vs. 28.9%, *p*<0.001), compared to non-users. Sixty-seven percent of the sample reported having public health insurance (Medicare and/or Medicaid), but PWUD were more likely than non-users to report no insurance or not knowing (25.3% vs. 16.3%, *p*<0.001). In addition, both PWUD and non-users had similar incomes, approximately two-thirds of both groups reported an annual income of less than $15,000, which reflects the social vulnerability of this sample (Table 1).

### COVID-19 testing and positivity

Nearly 16.0% of participants had never been tested for COVID-19 prior to participation in the study. Of those who had been tested (84.2%), 19.3% reported ever testing positive. Of those ever testing positive, 68.9% reported having moderate-to-severe symptoms, and 19.7% reported being hospitalized due to COVID-19. While there was no difference in the proportions of those who self- reported ever being tested for COVID-19 (83.3% in PWUD vs. 84.6% in non-users, *p* = 0.499), PWUD were less likely than non-users to self-report ever testing positive (14.7% vs. 21.0%, *p* = 0.006). However, 2.6% of participants tested positive for SARS-CoV-2, which tended to be more frequent among PWUD than non-users (3.7% vs. 2.2%, *p* = 0.076) (Table 2). Of eligible participants, 108 (5.7%) failed to arrive for COVID-19 testing, however the proportions of PWUD and non-users who failed to complete COVID-19 testing were not significantly different (6.3% vs. 5.5%, *p* = 0.495).

**Table 2:** COVID-19 testing and related beliefs and attitudes among RADx-UP study participants by drug use status, Miami, Florida, March 2021 –October 2022.

Prompt ^a^	Response	Total	Non-Users	People Who Use Drugs ^b^	*p*
Total		n = 1,780	n = 1,290 (72.5)	n = 490 (27.5)	
Have you ever been tested for COVID-19?	Yes	1,499 (84.2)	1,091 (84.6)	408 (83.3)	0.499
Have you ever tested positive for COVID-19? (n = 1,499) ^c^	Yes	289 (19.3)	229 (21.0)	60 (14.7)	0.006 *
How severe was your COVID-19 infection? (n = 289) ^d^	Moderate/Severe	199 (68.9)	161 (70.3)	38 (63.3)	0.299
Have you been hospitalized due to COVID-19? (n = 289) ^d^	Yes	57 (19.7)	45 (20.0)	12 (20.0)	0.952
“Someone close to me has tested positive for a COVID-19 infection”	True	994 (55.8)	707 (54.8)	287 (58.6)	0.153
“I do not know anyone who has tested positive for COVID-19 infection”	True	583 (32.8)	428 (33.2)	155 (31.6)	0.535
**Study testing**					
rt-PCR for SARS-CoV-2	Positive	46 (2.6)	28 (2.2)	18 (3.7)	0.076
**Access to testing**					
“I know where I can get COVID-19 testing in my community.”	Agree/Strongly agree	1,572 (88.3)	1,133 (87.8)	439 (89.6)	0.301
“It is easy to get tested for COVID-19.”	Agree/Strongly agree	1,553 (87.3)	1,124 (87.1)	429 (87.6)	0.813
Have you ever tried to get tested for COVID-19 but were unsuccessful?	Yes	142 (8.0)	92 (7.1)	50 (10.2)	0.033 *
**Perceived accuracy of testing**					
How confident are you that a negative test result means that you do not have COVID-19?	Confident/Very confident	1,446 (81.2)	1,065 (82.6)	381 (77.8)	0.020 *
How confident are you that a positive test result means that you do have COVID-19?	Confident/Very confident	1,218 (68.5)	898 (69.7)	320 (65.3)	0.077
**Perceived benefits of testing**					
*How much do the following encourage you to get tested*?					
Reduce worry that I might have COVID-19.	Somewhat or more	1,473 (83.1)	1,066 (83.1)	407 (83.1)	0.990
Believe that I was exposed to someone who has COVID-19.	Somewhat or more	1,484 (83.6)	1,078 (83.9)	406 (82.9)	0.599
To know if I am safe not to give COVID-19 to friends and family.	Somewhat or more	1,593 (89.8)	1,149 (89.4)	444 (90.8)	0.390
To know if I am safe not to give COVID-19 to anyone I am around.	Somewhat or more	1,597 (90.0)	1,153 (89.7)	444 (90.8)	0.475
To let my employer know that I am safe to work.	Somewhat or more	1,513 (85.0)	1,093 (84.7)	420 (85.7)	0.603
To get treated early (if I am positive).	Somewhat or more	1,664 (93.8)	1,205 (93.9)	459 (93.7)	0.892
**Perceived risks of testing**					
*How much do the following discourage you to get tested*?					
May experience discomfort from being tested.	Somewhat or more	298 (16.8)	199 (15.5)	99 (20.3)	0.016 *
Even if I don’t have it when tested, I can still get COVID-19 later.	Somewhat or more	317 (17.9)	217 (16.9)	100 (20.4)	0.082
I don’t have COVID-19 symptoms so I don’t need to be tested.	Somewhat or more	397 (22.4)	286 (22.3)	111 (22.7)	0.854
If I’m positive, officials will need to contact the people I’ve been in contact with.	Somewhat or more	301 (17.0)	213 (16.6)	88 (18.0)	0.476
I don’t want to know if I have it.	Somewhat or more	180 (10.1)	130 (10.1)	50 (10.2)	0.935
Not much they can do for me if I have it.	Somewhat or more	214 (12.0)	150 (11.7)	64 (13.1)	0.416
Difficult to get needed healthcare if I have it.	Somewhat or more	188 (10.6)	123 (9.6)	65 (13.3)	0.023 *
**Interpretation of negative or positive results**					
**If I get a negative test result, it means…**	I don’t have to worry about getting COVID-19	270 (15.2)	193 (15.0)	77 (15.7)	0.692
	I don’t have COVID-19 now	1,425 (80.1)	1,041 (80.7)	384 (78.4)	0.272
	I can be around others without giving the virus to them	708 (39.8)	516 (40.0)	192 (39.2)	0.753
	I can be around others without getting the virus from them	239 (13.4)	171 (13.3)	68 (13.9)	0.731
**If I get a positive result, it means…**	I will need to be admitted to the hospital	774 (43.5)	523 (40.5)	251 (51.2)	<0.001 *
	I will need to isolate myself from others	1,696 (95.3)	1,228 (95.2)	468 (95.5)	0.779
	I will need to take off work	1,272 (71.5)	877 (68.0)	395 (80.6)	<0.001 *
**Perceptions**					
How serious do you think it would be if you got COVID-19?	Moderately/Very	994 (55.8)	678 (52.6)	316 (64.5)	<0.001 *

Data are presented as count (percent, %).

Reported use of cannabis, cocaine or crack, heroin, fentanyl, methamphetamine, hallucinogens, ecstasy, or misuse of prescription drugs in the past 12 months.

Item asked to participants who reported having ever been tested for COVID-19.

Item asked to participants who reported having ever tested positive for COVID-19.

* p-value <0.05

Abbreviations: RADx-UP, Rapid Acceleration of Diagnostics–Underserved Populations; rt-PCR, reverse transcription-polymerase chain reaction

### Attitudes and beliefs about COVID-19 testing

There were no significant differences in the proportions of those who self- reported ever being tested for COVID-19 by drug use. However, when asked, “Have you ever tried to get tested for COVID-19 but were unsuccessful?” a greater proportion of PWUD reported, “yes” than non-users (10.2% vs. 7.1% respectively, *p* = 0.033), indicating a lack of testing access. The majority of participants reported being confident or very confident about COVID-19 testing results, but fewer trusted a positive (68.5%) than a negative (81.2%) result. On the other hand, PWUD were less likely than non-users to trust a negative result (77.8% vs. 82.6%, *p* = 0.020). When asked about perceived benefits of COVID-19 testing, over 80% of participants agreed with all prompts (Table 2). However, when asked about perceived risks of being tested, PWUD were more likely than non-users to report, “May experience discomfort from being tested” (20.3% vs. 15.5%, *p* = 0.016) and, “Difficult to get needed healthcare if I have it” (13.3% vs. 9.6%, *p* = 0.023). When asked about what a positive result meant, PWUD were more likely than non-users to report, “I will need to be admitted to the hospital” (51.2% vs. 40.5%, *p*<0.001) and, “I will need to take off work” (80.6% vs. 68.0%, *p*<0.001). When asked how serious they thought it would be if they got COVID-19, PWUD were more likely than non-users to report moderately-to-very serious (64.5% vs. 52.6%, *p*<0.001) (Table 2).

### COVID-19 vaccinations

Nearly 76% of the sample (75.8%) had received at least one dose of a COVID-19 vaccine (Table 3). PWUD, compared to non-users, were less likely to have received a flu vaccine that season (40.1% vs. 47.5%, *p* = 0.019) and any dose of a COVID-19 vaccine (65.5% vs. 79.7%, *p*<0.001) (Table 3). Multiple binary logistic regression estimated that PWUD had 37% lower odds of being vaccinated against COVID-19 compared to non-users after adjustment for age, sex, race/ethnicity, education, and history of lung disease (aOR, 0.63; 95% CI, 0.49–0.81; *p*<0.001) (Table 4). When stratified by type of drug used, use of cannabis was associated with 32% lower odds of being vaccinated (aOR, 0.68; 95% CI, 0.52–0.88; p = 0.003), and illicit drug use excluding cannabis (cocaine or crack, heroin, fentanyl, methamphetamine, hallucinogens, or ecstasy) was associated with 47% lower odds of being vaccinated (aOR, 0.53; 95% CI, 0.37–0.75; p<0.001). Due to the small proportions (0.4%-0.9%) of those who reported use of drugs other than cannabis or cocaine/crack (Fig 2), we were unable to conduct analyses stratified by each drug type separately. Of those participants who had received at least one dose of a COVID-19 vaccine, PWUD were less likely to have completed the initial vaccination series (90.2% vs. 94.0%, *p* = 0.026), compared to non-users (Table 3). Additionally, PWUD were also more likely to have refused a vaccine in the past (COVID-19 vaccines among other vaccines) (20.2% vs. 12.8%, *p*<0.001), compared to non-users. Among those who were unvaccinated against COVID-19 (n = 426), 49.5% reported not being likely to vaccinate and a greater proportion of PWUD reported they were not likely to vaccinate compared to non-users (58.9% vs. 43.4%, *p* = 0.002) (Table 3).

**Table 3:** COVID-19 vaccinations and related beliefs and attitudes among RADx-UP study participants by drug use status, Miami, Florida, March 2021 –October 2022.

Prompt ^a^	Response	Total	Non-Users	People Who Use Drugs ^b^	*p*
Total		n = 1,780	n = 1,290 (72.5)	n = 490 (27.5)	
Have you ever received a flu vaccination?	Yes	1,243 (69.8)	901 (69.8)	342 (69.8)	0.984
Have you received a flu vaccine this season? (n = 1,243) ^c^	Yes	565 (45.5)	428 (47.5)	137 (40.1)	0.019 *
Have you refused vaccination of a certain type of vaccine in the past?	Yes	264 (14.9)	165 (12.8)	99 (20.2)	<0.001 *
Have you received a COVID-19 vaccine?	Yes	1,349 (75.8)	1,028 (79.7)	321 (65.5)	<0.001 *
Have you completed the COVID-19 vaccination course? (n = 1,273) ^d^	Yes	1,185 (93.1)	918 (94.0)	267 (90.2)	0.026 *
Have you received a booster dose of a COVID-19 vaccine? (n = 559) ^e^	Yes	172 (30.8)	146 (32.2)	26 (24.5)	0.122
How likely are you to get an approved COVID-19 vaccine? (n = 426) ^f^	Not too likely/Not at all likely/Definitely not/ Don’t know	210 (49.5)	111 (43.4)	99 (58.9)	0.002 *
***Reasons for Getting/Not Getting a COVID 19 Vaccine***					
Why would you get a COVID-19 vaccine?	I want to keep my family safe	1,243 (69.8)	916 (71.0)	327 (66.7)	0.079
	I want to keep my community safe	1,075 (60.4)	780 (60.5)	295 (60.2)	0.920
	I want to keep myself safe	1,478 (83.0)	1,087 (84.3)	391 (79.8)	0.025 *
	I have a chronic health problem, like asthma or diabetes	583 (32.8)	434 (33.6)	149 (30.4)	0.194
	My doctor told me to get a COVID-19 vaccine	625 (35.1)	439 (34.0)	186 (38.0)	0.121
	I don’t want to get really sick from COVID-19	1,160 (65.2)	856 (66.4)	304 (62.0)	0.088
	I want to feel safe around other people	1,079 (60.6)	771 (59.8)	308 (62.9)	0.233
	I believe life won’t go back to normal until most people get a COVID-19 vaccine	774 (43.5)	550 (42.6)	224 (45.7)	0.242
Why would you NOT get a COVID-19 vaccine?	I’m allergic to vaccines	101 (5.7)	72 (5.6)	29 (5.9)	0.784
	I don’t like needles	160 (9.0)	104 (8.1)	56 (11.4)	0.027 *
	I’m not concerned about getting really sick from COVID-19	104 (5.8)	61 (4.7)	43 (8.8)	0.001 *
	I’m concerned about side effects from the vaccine	659 (37.0)	456 (35.4)	203 (41.4)	0.018 *
	I don’t think vaccines work very well	212 (11.9)	132 (10.2)	80 (16.3)	<0.001 *
	I don’t trust that the vaccine will be safe	462 (26.0)	297 (23.0)	165 (33.7)	<0.001 *
	I don’t believe the COVID-19 pandemic is as bad as some people say it is	76 (4.3)	42 (3.3)	34 (6.9)	0.001 *
	I don’t want to pay for it	130 (7.3)	73 (5.7)	57 (11.6)	<0.001 *
	I don’t know enough about how well a COVID-19 vaccine works	495 (27.8)	320 (24.8)	175 (35.7)	<0.001 *
“COVID-19 vaccination is an effective way to prevent and control COVID-19” (n = 1,681) ^g^	Agree/Strongly agree	1,483 (88.5)	1,094 (89.5)	389 (85.8)	0.041 *
Which of these statements best describes the most important reason for not choosing to get vaccinated: (n = 1,700)	I do not believe the vaccine is safe	385 (22.7)	268 (21.7)	117 (25.2)	<0.001 *
	I do not believe the vaccine is effective	95 (5.6)	65 (5.3)	30 (6.5)	
	I do not trust the source that encouraged me to get the vaccine	32 (1.9)	14 (1.1)	18 (3.9)	
	I do not believe in ANY vaccines, & my reason is not any different for a new COVID-19 vaccine	19 (1.1)	14 (1.1)	5 (1.1)	
	A source that I trust encouraged me to NOT get the vaccine	15 (0.9)	8 (0.7)	7 (1.5)	
	I don’t have a strong feeling about the vaccine and probably won’t get it	56 (3.3)	33 (2.7)	23 (5.0)	
	I already got COVID-19 infection, so I don’t think I need it	30 (1.8)	23 (1.9)	7 (1.5)	
“The side effects of most vaccines outweigh the benefits”	Agree/Strongly agree	536 (30.1)	387 (30.0)	149 (30.4)	0.867
“If I decide to get the COVID-19 vaccine, it would be hard to find a provider or clinic that could give me the vaccine.”	Agree/Strongly agree	204 (11.5)	166 (12.9)	38 (7.8)	0.003 *

Data are presented as count (percent, %).

Raeported use of cannabis, cocaine or crack, heroin, fentanyl, methamphetamine, hallucinogens, ecstasy, or misuse of prescription drugs in the past 12 months.

Item asked to participants who reported ever receiving a flu vaccination.

Item asked during Phase I to those participants who reported they have received a COVID-19 vaccine. Vaccination items were added to and/or removed from the survey as vaccinations and guidelines were developed and recommendations were updated by the Centers for Disease Control and Prevention (CDC).

Item asked to a subset of Phase I participants and all of Phase II participants who reported they have received a COVID-19 vaccine. Vaccination items were added to and/or removed from the survey as vaccinations and guidelines were developed and recommendations were updated by the CDC.

Item asked to participants who reported they have not received a COVID-19 vaccine.

Item only asked during Phase I.

* p-value <0.05

Abbreviations: RADx-UP, Rapid Acceleration of Diagnostics–Underserved Populations

**Table 4:** Univariate and multiple binary logistic regressions for COVID-19 vaccination status and COVID-19 positivity by drug use status, Miami, Florida, March 2021 –October 2022.

Variable	Unadjusted OR	95% CI	*p*	Adjusted OR ^a^^,^^b^	95% CI	*p*
**Vaccinated for COVID-19** ^c^						
Any drug use ^d^	0.48	0.38–0.61	<0.001 *	0.63	0.49–0.81	<0.001 *
Cannabis use ^e^	0.49	0.39–0.63	<0.001 *	0.68	0.52–0.88	0.003 *
Illicit drug use ^f^	0.49	0.35–0.68	<0.001 *	0.53	0.37–0.75	<0.001 *
Misuse of prescription drugs ^g^	0.70	0.24–2.03	0.511	0.92	0.29–2.92	0.893
**Tested positive for SARS-CoV-2 via rt-PCR testing**						
Any drug use	1.71	0.94–3.13	0.079	1.57	0.81–3.04	0.179
Cannabis use	1.96	1.06–3.60	0.031 *	1.72	0.89–3.36	0.110
Illicit drug use	1.23	0.48–3.17	0.663	1.37	0.52–3.65	0.527
Misuse of prescription drugs ^h^	-	-	-	-	-	-

Multivariable regression models adjusted for age, sex, race/ethnicity, education level, and history of lung disease. COVID-19 vaccination status was also included as a covariate for the logistic regression models with COVID-19 positivity as the outcome.

The variance inflation factor method was used to ensure absence of collinearity between independent variables.

Received ≥1 dose of a COVID-19 vaccine.

Reported use of cannabis, cocaine or crack, heroin, fentanyl, methamphetamine, hallucinogens, ecstasy, or misuse of prescription drugs in the past 12 months.

Responded “yes” to “Have you used marijuana in the past 12 months?”.

Responded “yes” to “In the past 12 months, have you used any of the following drugs: cocaine or crack, heroin, fentanyl, crystal meth (methamphetamine), hallucinogens (like LSD, psilocybin, PCP, ketamine), ecstasy?”.

Responded “About once per month”, “About once or twice per week”, or “Daily or almost daily” to “In the past 12 months, how often have you used prescription drugs just for the feeling, more than prescribed, or that were not prescribed for you?”.

Unable to interpret OR due to small cell counts caused by rare outcome event rate and small proportion of those who reported prescription drug misuse (n = 46 tested positive for SARS-CoV-2 via rt-PCR testing and n = 16 reported prescription drug misuse).

* p-value <0.05

### Reasons for getting/not getting a COVID-19 vaccine

While 88.5% agreed with, “COVID-19 vaccination is an effective way to prevent and control COVID-19”, 30.1% agreed with, “The side effects of most vaccines outweigh the benefits” (Table 3). Notably, PWUD were less likely than non-users to agree with, “COVID-19 vaccination is an effective way to prevent and control COVID-19” (85.8% vs. 89.5%, *p* = 0.041). PWUD largely agreed with non-users on reasons for getting a COVID-19 vaccine, but PWUD were less likely to report, “I want to keep myself safe” (79.8% vs. 84.3%, *p* = 0.025). In terms of reasons for not getting a COVID-19 vaccine, PWUD, compared to non-users, were more likely to report, “I don’t like needles” (11.4% vs. 8.1%, *p* = 0.027), “I’m not concerned about getting really sick from COVID-19” (8.8% vs. 4.7%, *p* = 0.001), “I’m concerned about side effects from the vaccine” (41.4% vs. 35.4%, *p* = 0.018), “I don’t think vaccines work very well” (16.3% vs. 10.2%, *p*<0.001), “I don’t trust that the vaccine will be safe” (33.7% vs. 23.0%, *p*<0.001), “I don’t believe the COVID-19 pandemic is as bad as some people say it is” (6.9% vs. 3.3%, *p* = 0.001), “I don’t want to pay for it” (11.6% vs. 5.7%, *p*<0.001), and, “I don’t know enough about how well a COVID-19 vaccine works” (35.7% vs. 24.8%, *p*<0.001). Additionally, PWUD, compared to non-users, were less likely to agree with, “If I decide to get the COVID-19 vaccine, it would be hard to find a provider that could give me the vaccine” (7.8% vs. 12.9%, *p* = 0.003).

## Discussion

People who use drugs are a socially vulnerable population [1–3] who experience high rates of poverty [5], housing instability, and difficulty accessing healthcare [6]. Taken together with the underlying cardiometabolic comorbidities and pulmonary damage caused by illicit substances [28–31] as well as the immunosuppressive properties that characterize a wide range of drugs [32, 33], PWUD have the potential to be greatly impacted by the COVID-19 pandemic. We explored relationships between drug use, COVID-19 testing, vaccination, and infection and found that PWUD were more likely, compared to non-users, to experience adversity brought about by the pandemic, such as housing insecurity and loss of income. This study also provides evidence that PWUD face disparities in COVID-19 testing access, and to the best of our knowledge, is one of the first to report lower odds of COVID-19 vaccination among PWUD in the Southern U.S. compared to non-using peers. While there were no significant differences in the proportions of those who self-reported ever being tested for COVID-19 by drug use, PWUD were more likely to face difficulties accessing testing. Interestingly, PWUD, compared to non-users, were less likely to self-report ever testing positive. However, SARS-CoV-2 positivity during the study tended to be more frequent among PWUD than non-users. Importantly, PWUD had lower odds of being vaccinated compared to non-users after adjustment for covariates. These findings highlight the need for improved efforts tailored to PWUD to promote better access to and utilization of testing and vaccination during the COVID-19 pandemic and future crises.

Testing for active viral disease is a cornerstone of contagion containment strategies. With one in six participants who had never been tested prior to participation in this study more than a year into the pandemic, understanding barriers to testing is critical to increasing testing uptake. Our data show that PWUD, more so than non-users, had tried to get tested for COVID-19 but were unsuccessful, were less confident in the accuracy of a negative result, and felt it would be difficult to get needed healthcare if positive. Altogether this indicates that PWUD may have faced a lack of access to testing and were discouraged from being tested due to a perceived lack of accuracy of the results and concerns about healthcare access. Distrust in the medical and research systems due to breaches of trust, mistreatment, and disclosure of protected health information in PWUD could also play a role in discouragement from COVID-19 testing [44]. Our findings agree with those in the literature that found COVID-19 testing was not broadly accessed by drug users and other marginalized populations [45]. However, most studies were conducted mainly in injection drug users [45, 46], and found that less than a third had ever been tested [47]. Our study differs from this literature in that a very small proportion of our participants reported injecting drugs (1.4% of the MASH cohort). Thus, we explored COVID-19 testing in relation to drug use of any type by any route of administration and indeed found disparities in difficulties accessing testing.

In terms of the potential increased risk for COVID-19 that PWUD face, Wang et al., found that a recent diagnosis of a substance use disorder (SUD) was associated with increased risk of COVID-19, hospitalization, and mortality [29]. Additionally, people with a recent SUD, compared to those without, had higher rates of morbidities related to worse COVID-19 outcomes (i.e., chronic lung and cardiovascular diseases, etc.). We found that PWUD were actually less likely to self-report prior COVID-19 illness, but we did not observe a significant difference in SARS-CoV-2 positivity via rt-PCR, although it tended to be more frequent among PWUD than non-users. We also found no associations between drug use and COVID-19 symptom severity or hospitalizations, but PWUD were more likely than non-users to expect worse COVID-19 outcomes. Our finding of no significant difference in SARS-CoV-2 positivity could be explained by the low rate of positivity observed; less than 3% tested positive. This could be due to the community-based study setting rather than a healthcare facility as well as the participants’ discomfort with the need to quarantine and a potential loss of income. Thus, participants may not have presented for testing when experiencing symptoms.

COVID-19 vaccines have been shown to be effective at preventing severe illness, hospitalization, and death due to COVID-19 [10]. Yet, we found that PWUD had 37% lower odds of being vaccinated compared to non-users after adjustment for covariates. Studies assessing self-reported injection drug use have also reported lower vaccination rates compared to non-users [18, 21]. It is unclear whether the lower vaccination rate among PWUD is due to poor access, vaccine hesitancy, or a combination of the two. However, we found that PWUD were more likely, compared to non-users, to report, “I don’t like needles”, “I’m not concerned about getting really sick from COVID-19”, “I’m concerned about side effects from the vaccine”, “I don’t think vaccines work very well”, “I don’t trust that the vaccine will be safe”, “I don’t believe the COVID-19 pandemic is as bad as some people say it is”, and, “I don’t know enough about how well a COVID-19 vaccine works”. Additionally, when unvaccinated participants were asked how likely they were to get a COVID-19 vaccine, PWUD were more likely than non-users to respond with, “don’t know”, “not too likely”, “not at all likely”, or, “definitely not”, suggesting that hesitancy could in fact, be a main driver of this disparity. Additionally, PWUD were more likely, compared to non-users, to have refused a vaccine in the past (COVID-19 vaccines among other vaccines). This supports the notion that drug use may be associated with hesitancy of other types of vaccines [22–26]. Although COVID-19 vaccines were, at the time, provided free-of-charge in the U.S., PWUD were more likely to report, “I don’t want to pay for it” than non-users, suggesting that immunization campaigns advertising vaccines as free regardless of insurance status [48], may not be reaching or be trusted by PWUD.

This research highlights the need for testing and immunization plans that are tailored to PWUD who are likely to face vaccine hesitancy and social barriers to testing, such as lack of transportation and technology, as well as competing priorities in the form of housing and food insecurity [49]. Therefore, effective strategies are needed to overcome testing barriers and vaccination hesitancy for this vulnerable population during the COVID-19 pandemic and future crises. Strengths of this study include the use of a large sample of underserved, low-income, racial/ethnic minority adults from Miami, Florida which experiences a high level of social vulnerability [37, 38] and our ability to confirm over 80% of all self-reported COVID-19 vaccinations with medical records. Additionally, as part of RADx-UP, future studies with larger sample sizes from cohorts across the nation are possible. Limitations for this research include the use of a sample that may not be representative of PWUD in Miami, self-report of substance use which is susceptible to recall bias and underreporting, and 12-month timeframe of drug use which may also contribute to recall bias. We do note, however, that previous studies have been successful in utilizing self-reported substance use data during the COVID-19 pandemic [18, 21, 47]. The cross-sectional design also does not allow for causality or temporality to be established (i.e., we cannot determine if drug use influenced COVID-19 outcomes, if the pandemic influenced drug use behaviors, or if the relationship is bidirectional). Finally, we were also unable to further stratify our sample by type of illicit drug used because of the small proportions (<1%) of participants who reported drug use other than cannabis or cocaine/crack.

## Conclusion

PWUD presented with greater difficulties accessing COVID-19 testing and more concerns regarding testing accuracy and perceived risks of being tested. Compared to non-users, PWUD presented with greater vaccine hesitancy and significantly lower odds of COVID-19 vaccination. Testing and immunization plans that are tailored to the needs of PWUD and consider access, trust-building campaigns, and education may be needed during the COVID-19 pandemic and future crises.

## Supporting information

S1 Checklist STROBE statement—checklist of items that should be included in reports of *cross-sectional studies*. (DOC)

S1 Data (XLSX)
