# Supplementary material for: Drug use and COVID-19 testing, vaccination, and infection among underserved, minority communities in Miami, Florida
Source: PLoS One. 2024 Apr 30;19(4):e0297327. doi: 10.1371/journal.pone.0297327 (PMC11060546; doi:10.1371/journal.pone.0297327)
Supplement: S1 Checklist — (DOC) [file pone.0297327.s001.doc]

STROBE Statement—Checklist of items that should be included in reports of ***cross-sectional studies***

|  | Item No | Recommendation |
| --- | --- | --- |
| **Title and abstract** | 1 | (*a*) Indicate the study’s design with a commonly used term in the title or the abstract – completed (see text line 26) |
|  |  | (*b*) Provide in the abstract an informative and balanced summary of what was done and what was found – completed (see page 1) |
| Introduction | | |
| Background/rationale | 2 | Explain the scientific background and rationale for the investigation being reported – completed (see page 2-3) |
| Objectives | 3 | State specific objectives, including any prespecified hypotheses – completed (see text lines 88-89) |
| Methods | | |
| Study design | 4 | Present key elements of study design early in the paper – completed (see page 4-7) |
| Setting | 5 | Describe the setting, locations, and relevant dates, including periods of recruitment, exposure, follow-up, and data collection – completed (see page 4-7) |
| Participants | 6 | (*a*) Give the eligibility criteria, and the sources and methods of selection of participants – completed (see text lines 123-125 and 99-109) |
| Variables | 7 | Clearly define all outcomes, exposures, predictors, potential confounders, and effect modifiers. Give diagnostic criteria, if applicable – completed (see text lines 172-177) |
| Data sources/ measurement | 8* | For each variable of interest, give sources of data and details of methods of assessment (measurement). Describe comparability of assessment methods if there is more than one group – completed (see pages 4-7) |
| Bias | 9 | Describe any efforts to address potential sources of bias – completed (see text lines 121-122 and 452-455) |
| Study size | 10 | Explain how the study size was arrived at – completed (see text lines 185-189 and Fig 1) |
| Quantitative variables | 11 | Explain how quantitative variables were handled in the analyses. If applicable, describe which groupings were chosen and why – completed (see page 7) |
| Statistical methods | 12 | (*a*) Describe all statistical methods, including those used to control for confounding – completed (see pages 7) |
|  |  | (*b*) Describe any methods used to examine subgroups and interactions – completed (see pages 7) |
|  |  | (*c*) Explain how missing data were addressed – completed (see text lines 178-179) |
|  |  | (*d*) If applicable, describe analytical methods taking account of sampling strategy – N/A |
|  |  | (*e*) Describe any sensitivity analyses – N/A |
| Results | | |
| Participants | 13* | (a) Report numbers of individuals at each stage of study—eg numbers potentially eligible, examined for eligibility, confirmed eligible, included in the study, completing follow-up, and analysed – completed (see text lines 185-189 and Fig 1) |
|  |  | (b) Give reasons for non-participation at each stage – completed (see text lines 185-189 and Fig 1) |
|  |  | (c) Consider use of a flow diagram – completed (see Fig 1) |
| Descriptive data | 14* | (a) Give characteristics of study participants (eg demographic, clinical, social) and information on exposures and potential confounders – completed (see pages 8-11 and Table 1) |
|  |  | (b) Indicate number of participants with missing data for each variable of interest – completed (see rows and footnotes on Tables 1 – 3) |
| Outcome data | 15* | Report numbers of outcome events or summary measures – completed (see text lines 189-192 and Fig 2) |
| Main results | 16 | (*a*) Give unadjusted estimates and, if applicable, confounder-adjusted estimates and their precision (eg, 95% confidence interval). Make clear which confounders were adjusted for and why they were included – completed (see Table 4) |
|  |  | (*b*) Report category boundaries when continuous variables were categorized – completed (see Table 1) |
|  |  | (*c*) If relevant, consider translating estimates of relative risk into absolute risk for a meaningful time period – N/A |
| Other analyses | 17 | Report other analyses done—eg analyses of subgroups and interactions, and sensitivity analyses – N/A |
| Discussion | | |
| Key results | 18 | Summarise key results with reference to study objectives – completed (see page 19) |
| Limitations | 19 | Discuss limitations of the study, taking into account sources of potential bias or imprecision. Discuss both direction and magnitude of any potential bias – completed (see text lines 456-465) |
| Interpretation | 20 | Give a cautious overall interpretation of results considering objectives, limitations, multiplicity of analyses, results from similar studies, and other relevant evidence – completed (see text lines 467-473) |
| Generalisability | 21 | Discuss the generalisability (external validity) of the study results – completed (see text lines 456-457) |
| Other information | | |
| Funding | 22 | Give the source of funding and the role of the funders for the present study and, if applicable, for the original study on which the present article is based – completed (submitted via PLOS One submission portal) |

*Give information separately for exposed and unexposed groups.

**Note:** An Explanation and Elaboration article discusses each checklist item and gives methodological background and published examples of transparent reporting. The STROBE checklist is best used in conjunction with this article (freely available on the Web sites of PLoS Medicine at http://www.plosmedicine.org/, Annals of Internal Medicine at http://www.annals.org/, and Epidemiology at http://www.epidem.com/). Information on the STROBE Initiative is available at www.strobe-statement.org.
